# Simultaneous knockout of VITAMIN C DEFECTIVE 2 and 3 exacerbates ascorbate deficiency and light stress sensitivity

**DOI:** 10.1093/jxb/eraf352

**Published:** 2025-08-06

**Authors:** Yasuhiro Tanaka, Takanori Maruta, Koki Arima, Hiroki Nakayama, Akane Hamada, Takahiro Ishikawa

**Affiliations:** Graduate School of Natural Science and Technology, Shimane University, 1060 Nishikawatsu, Matsue, Shimane 690-8504, Japan; Bioresource and Life Sciences, The United Graduate School of Agricultural Sciences, Tottori University, 4-101 Koyama-Minami, Tottori, Tottori 680-8553, Japan; Graduate School of Natural Science and Technology, Shimane University, 1060 Nishikawatsu, Matsue, Shimane 690-8504, Japan; Bioresource and Life Sciences, The United Graduate School of Agricultural Sciences, Tottori University, 4-101 Koyama-Minami, Tottori, Tottori 680-8553, Japan; Department of Life Sciences, Faculty of Life and Environmental Science, Shimane University, 1060 Nishikawatsu, Matsue, Shimane 690-8504, Japan; Institute of Agricultural and Life Sciences, Academic Assembly, Shimane University, 1060 Nishikawatsu, Matsue, Shimane 690-8504, Japan; Department of Life Sciences, Faculty of Life and Environmental Science, Shimane University, 1060 Nishikawatsu, Matsue, Shimane 690-8504, Japan; Department of Life Sciences, Faculty of Life and Environmental Science, Shimane University, 1060 Nishikawatsu, Matsue, Shimane 690-8504, Japan; Graduate School of Natural Science and Technology, Shimane University, 1060 Nishikawatsu, Matsue, Shimane 690-8504, Japan; Bioresource and Life Sciences, The United Graduate School of Agricultural Sciences, Tottori University, 4-101 Koyama-Minami, Tottori, Tottori 680-8553, Japan; Graduate School of Natural Science and Technology, Shimane University, 1060 Nishikawatsu, Matsue, Shimane 690-8504, Japan; Bioresource and Life Sciences, The United Graduate School of Agricultural Sciences, Tottori University, 4-101 Koyama-Minami, Tottori, Tottori 680-8553, Japan; Department of Life Sciences, Faculty of Life and Environmental Science, Shimane University, 1060 Nishikawatsu, Matsue, Shimane 690-8504, Japan; Institute of Agricultural and Life Sciences, Academic Assembly, Shimane University, 1060 Nishikawatsu, Matsue, Shimane 690-8504, Japan; VIB-Ghent University, Belgium

**Keywords:** *Arabidopsis thaliana*, ascorbate biosynthesis, GDP-L-galactose phosphorylase, high light, oxidative stress, vitamin C-defective

## Abstract

Light-dependent activation of ascorbate biosynthesis is essential for accumulating ascorbate to mitigate photooxidative stress. The *VTC2* and *VTC3* genes play key roles in this process: *VTC2* encodes GDP-L-galactose phosphorylase (GGP), the rate-limiting enzyme in ascorbate biosynthesis, and *VTC3* encodes a putative protein kinase/phosphatase with an unknown function. Here, we investigated their functional and genetic relationship. In *vtc3* mutants, *VTC2* transcription and GGP activity were slightly enhanced, suggesting that VTC3 is not required for *VTC2* expression. Additionally, the *vtc3* mutation had negligible effects on the transcriptome and the activity of enzymes involved in ascorbate redox cycle regulation, narrowing down the possible roles of VTC3 in ascorbate metabolism. Under low-light conditions, ascorbate concentrations were lower in *vtc2* than in *vtc3* mutants, but *vtc2* retained the ability to increase ascorbate concentrations under high-light stress, unlike *vtc3*. The simultaneous knockout of VTC2 and VTC3 further reduced ascorbate concentrations compared with the single mutants and severely impaired light-stress-induced ascorbate accumulation, resulting in impaired non-photochemical quenching and enhanced photooxidative damage. These findings highlight the additive effects of VTC2 and VTC3 on ascorbate biosynthesis and stress tolerance. The *vtc2 vtc3* double mutants provide a valuable model for further elucidating the physiological roles of ascorbate in plants.

## Teaser

VTC2 and VTC3 are crucial for activating ascorbate biosynthesis. Their simultaneous knockout exacerbates ascorbate deficiency under low- and high-light conditions, causing severe photooxidative damage.

## Introduction

In plants, reactive oxygen species (ROS) can be generated as byproducts of photosynthesis (Asada, 1999). Other major sources of ROS include the mitochondrial electron transport chain and the oxidation of glycolate in peroxisomes during photorespiration (Mittler *et al*., 2022). In addition, plants possess multiple apoplastic ROS-producing enzymes, particularly NADPH oxidases (also known as respiratory burst oxidase homologs) (Mittler *et al*., 2022). Under stress conditions, ROS generation through these various pathways is often inevitably increased or actively promoted as part of the plant’s response mechanisms. Although ROS are highly reactive and can damage cellular components at elevated concentrations, they also serve as critical signaling molecules in plant stress acclimation (Mittler *et al*., 2022). Maintaining ROS homeostasis is essential for harnessing their signaling functions without compromising cellular integrity. This balance is achieved through robust antioxidant systems, among which ascorbate is one of the most abundant soluble antioxidants in plant leaves, being present in all intracellular compartments at millimolar concentrations (Zechmann *et al*., 2011; Smirnoff, 2018; Maruta *et al*., 2024). Ascorbate plays a central role in scavenging ROS through both direct non-enzymatic reactions and enzymatic pathways mediated by ascorbate peroxidase (APX), an enzyme specific to eukaryotic photosynthetic organisms (Shigeoka *et al*., 2002; Maruta *et al*., 2016). Additionally, ascorbate serves as a substrate for violaxanthin de-epoxidase, an enzyme crucial for the xanthophyll cycle, which dissipates excess light energy as heat and limits ROS production during photosynthesis (Müller-Moulé *et al*., 2004). Beyond its role in ROS management, ascorbate is involved in diverse biological processes, including photosynthetic electron transport, iron uptake, cell division, anthocyanin accumulation, and hormone biosynthesis (Smirnoff, 2018).

Several pathways for ascorbate biosynthesis have been proposed; however, the D-mannose/L-galactose (D-Man/L-Gal) pathway, also known as the Smirnoff–Wheeler pathway, is widely recognized as the primary route (Wheeler *et al*., 1998; Smirnoff, 2018; Maruta, 2022). This notion is supported by substantial experimental evidence demonstrating its involvement in ascorbate biosynthesis across land plants and algae (Wheeler *et al*., 1998; Conklin *et al*., 1999; Vidal-Meireles *et al*., 2017; Sodeyama *et al*., 2021; Ishida *et al*., 2024). The D-Man/L-Gal pathway synthesizes ascorbate from the hexose phosphate pool through eight enzymatic reactions catalyzed sequentially by phosphomannose isomerase (PMI), phosphomannomutase (PMM), GDP-D-mannose pyrophosphorylase (GMP), GDP-D-mannose-3′,5′-epimerase (GME), GDP-L-galactose phosphorylase (GGP), L-galactose-1-phosphate phosphatase (GPP), L-galactose dehydrogenase (L-GalDH), and L-galactono-1,4-lactone dehydrogenase (L-GalLDH) (Smirnoff, 2018). The final step, catalyzed by L-GalLDH, occurs in the inner mitochondrial membrane, whereas all the other enzymatic steps take place in the cytosol (Smirnoff, 2018). The significance of the D-Man/L-Gal pathway has been confirmed mainly through the characterization of Arabidopsis vitamin C-defective (*vtc*) mutants. The genes *VTC1*, *VTC2*, and *VTC4* encode GMP, GGP, and GPP, respectively (Conklin *et al*., 1999, 2006; Jander *et al*., 2002; Laing *et al*., 2007; Linster *et al*., 2007). Arabidopsis also possesses a minor isoform of GGP, encoded by *VTC5*. Notably, a double knockout mutant of *VTC2* and *VTC5* results in undetectable ascorbate concentrations and exhibits seedling lethality unless supplemented with ascorbate or its precursors (Dowdle *et al*., 2007).

Light is a potent stimulus for increasing the size of the ascorbate pool in plants. Leaf ascorbate content rises in response to light intensity and exposure duration (Dowdle *et al*., 2007; Yabuta *et al*., 2007). The key enzyme regulating ascorbate content under light conditions is VTC2/GGP, whose transcription is induced by light (Dowdle *et al*., 2007; Yabuta *et al*., 2007). Interestingly, the light-dependent activation of *VTC2* transcription is suppressed by photosynthesis inhibitors, suggesting a role for photosynthesis in the regulation of ascorbate biosynthesis (Yabuta *et al*., 2007). GGP activity is significantly enhanced under high-light stress, whereas the activities of other biosynthetic enzymes are unaffected (Dowdle *et al*., 2007). Moreover, overexpression of VTC2 effectively enhances the ascorbate pool sizes in various plant species (Bulley *et al*., 2009, 2012; Yoshimura *et al*., 2014). These findings confirm that VTC2/GGP is the rate-limiting enzyme in the D-Man/L-Gal pathway, a conclusion further supported by computational simulations (Fenech *et al*., 2021). The regulation of VTC2 by light also occurs at the post-translational level. In the dark, GGP activity is inhibited by a new class of blue-light receptors, PAS/LOV proteins, which directly associate with GGP (Ogura *et al*., 2008; Aarabi *et al*., 2023; Bournonville *et al*., 2023). Upon exposure to blue light, PAS/LOV proteins dissociate from GGP, promoting its enzymatic activity. Thus, light-dependent activation of GGP plays a pivotal role in ascorbate biosynthesis. Recent studies have proposed that the biosynthetic enzymes in the cytosol form a protein complex (Fenech *et al*., 2021), potentially contributing to the regulation of ascorbate biosynthesis. Additionally, ascorbate itself tightly regulates its biosynthesis. High ascorbate concentrations repress VTC2 translation via an upstream open reading frame in the 5′-untranslated region of the gene, counterbalancing the light-induced activation of GGP (Laing *et al*., 2015). Consequently, ascorbate biosynthesis is finely tuned through transcriptional, translational, and post-translational regulation. However, the precise mechanisms underlying light-dependent activation of GGP remain to be elucidated.

The *VTC3* gene encodes a unique protein with both serine/threonine kinase and PP2C domains (Conklin *et al*., 2000, 2013). Two distinct *vtc3* alleles have been identified: *vtc3-1*, which carries a G202E missense mutation in the kinase domain, and *vtc3-2*, which has a nonsense mutation in the PP2C domain (Conklin *et al*., 2013). Both alleles exhibit a moderate ascorbate-deficient phenotype, with approximately 50% of wild-type ascorbate concentrations, a deficiency less severe than that observed in *vtc2* mutants (20–30% of wild-type concentrations). VTC3 is highly conserved across land plants and algae (Conklin *et al*., 2013; Maruta *et al*., 2024), and its loss also results in ascorbate deficiency in the moss *Physcomitrella patens* (Sodeyama *et al*., 2021). Notably, light-induced ascorbate accumulation is significantly impaired in Arabidopsis *vtc3* mutants (Conklin *et al*., 2013), suggesting that VTC3 plays a crucial role in the light regulation of VTC2/GGP. Since VTC3 has been reported to be a chloroplast protein, it is unlikely to regulate ascorbate biosynthesis through direct (de)phosphorylation of biosynthetic enzymes. The precise physiological function of VTC3 remains unclear, and further studies are required to elucidate its role.

The size of the ascorbate pool is regulated not only by its biosynthesis but also by its oxidation, recycling, and degradation. Reduced ascorbate (ASC) undergoes oxidation through APX or direct interaction with ROS, generating monodehydroascorbate (MDHA) (Asada, 1999; Smirnoff, 2018). MDHA radicals are disproportionated into ASC and dehydroascorbate (DHA). MDHA and DHA are recycled back to ASC by monodehydroascorbate reductase (MDAR) and dehydroascorbate reductase (DHAR), respectively (Foyer and Halliwell, 1977; Hossain and Asada, 1985). MDAR uses NAD(P)H, whereas DHAR relies on reduced glutathione (GSH) as the electron donor. Additionally, GSH can non-enzymatically reduce DHA to ASC (Winkler *et al*., 1994; Terai *et al*., 2020; Hamada *et al*., 2023). However, DHA is highly unstable and undergoes irreversible degradation if it is not rapidly reduced. These interconnected processes are essential for maintaining ascorbate homeostasis, and it remains possible that VTC3 regulates the ascorbate pool size by modulating ascorbate oxidation or degradation processes.

This study addresses two unresolved questions regarding the regulation of ascorbate homeostasis in plants. First, although VTC2/GGP is known to be a key regulatory enzyme in ascorbate biosynthesis and is strongly induced by light, the upstream signaling components controlling its activation remain poorly understood. Previous studies reported that *vtc3* mutants exhibit defective light-induced ascorbate accumulation (Conklin *et al*., 2013), suggesting a possible regulatory link between VTC3 and VTC2/GGP expression or activity. Based on this, we hypothesized that VTC3 facilitates the light-dependent activation of VTC2/GGP and thereby promotes ascorbate biosynthesis under light stress. Second, although *vtc2* and *vtc3* single mutants both show ascorbate deficiency, their distinct phenotypes raise the question of whether these genes act independently or synergistically. Notably, *vtc2* mutants retain the ability to increase their ascorbate concentrations under high-light stress, likely due to the residual function of the minor GGP isoform VTC5 (Terai *et al*., 2020; Hamada *et al*., 2023), whereas *vtc3* mutants fail to induce this response (Conklin *et al*., 2013). We therefore hypothesized that the simultaneous loss of both *VTC2* and *VTC3* would result in a more severe ascorbate-deficient phenotype and increased light-stress sensitivity. By characterizing single and double mutants of *vtc2* and *vtc3*, we aimed to test these hypotheses and gain new insights into the genetic and physiological control of ascorbate metabolism and photoprotection in Arabidopsis.

## Materials and methods

### Plant materials and growth conditions

*Arabidopsis thaliana* ecotype Col-0 was used as the wild type. The T-DNA insertion lines *vtc2-4* (SAIL_769_H05) and *vtc3-3* (SALK_095743) were obtained from the Arabidopsis Biological Resource Center and used for further crossings. Unless otherwise specified, wild-type and mutant seeds were sown in Jiffy-7 peat pellets and stratified in the dark at 4 °C for 2–3 days. Plants were grown in a growth chamber (KCLP-1000CCFL-6-8L-CO₂, NK System) at 22 °C under a 16 h photoperiod with low light intensity (40–60 µmol photons m^−2^ s^−1^) and at 20 °C in darkness for 8 h. At 3 weeks of age, plants were exposed to moderate-light (750 µmol photons m^−2^ s^−1^) or high-light (1500 µmol photons m^−2^ s^−1^) stress, using a light-emitting diode (LED) lighting unit (PFQ-600PT, NK System) without a preceding dark period. The LED system minimized heat production, maintaining leaf temperature at approximately 24–25 °C. To ensure uniform light exposure, plants were placed on a turntable beneath the LED panel. Fully expanded rosette leaves were collected from at least three plants and treated as a single biological replicate. Rosette and lesion areas (percentage of the total rosette area) were quantified using ImageJ software.

For ascorbate complementation assays, fully expanded leaves were excised from 3-week-old plants and floated on 20 mM MES buffer (pH 5.7) with or without 10 mM sodium ascorbate. The samples were incubated under continuous light for 24 h, followed by exposure to high-light conditions for an additional 24 h. Thereafter, leaves were subjected to trypan blue staining to visualize cell death. The stained area was quantified using ImageJ software.

For plate-grown experiments, wild-type and mutant plants were cultivated on half-strength Murashige and Skoog (MS) medium supplemented with 1% sucrose under a 16 h photoperiod at 100 µmol photons m^−2^ s^−1^ and 22 °C, with an 8 h dark period at 20 °C (LH-240S, NK System). Plants were then subjected to moderate-light stress (650 µmol photons m^−2^ s^−1^) without an intermediate dark period using the same LED panel and turntable system as described above. Shoots from at least 10 plants were pooled and treated as a single biological replicate.

### Semi-quantitative and quantitative reverse transcription–polymerase chain reaction

Semi-quantitative reverse transcription–polymerase chain reaction (RT–PCR) was performed according to Terai *et al*. (2020). Quantitative RT–PCR (qPCR) was performed as described by Iwagami *et al*. (2022). *Actin2* mRNA was used as an internal standard in all experiments. The expression stability of *Actin2* was confirmed by evaluating cycle threshold values across all experimental conditions. Three biological replicates per genotype/treatment combination were used, and each was derived from the average of three technical qPCR replicates. Expression variability was assessed using the coefficient of variation (CV). The consistently low CV values (ranging from 0.4% to 1.32%) support the use of *Actin2* as a stable reference gene under the tested conditions (see Supplementary Fig. S1). All the primers used are listed in Supplementary Table S1.

### Western blotting

To examine VTC2 protein expression, we attempted to generate two independent anti-VTC2 peptide antibodies. The peptide sequences used were NH_2_-C+QNSSGNVNQKSNRT-COOH and NH_2_-C+KRKEDYEGASEDNA-COOH. The design and synthesis of the antigenic peptides, as well as antibody production, were outsourced to Eurofins Genomics. In addition, we purchased a commercial anti-GDP-L-galactose phosphorylase antibody (AS10 723, Agrisera). Using these antibodies, we performed western blotting following the method described by Kameoka *et al*. (2021), but we were unable to detect the VTC2 protein.

### Measurements of antioxidants and enzyme activity

Ascorbate content was quantified using an ultra-fast liquid chromatography (UFLC) system equipped with a C18 column, following the method of Hamada and Maruta (2024). Glutathione concentrations were measured according to Noctor *et al*. (2016) without modifications. Soluble and membrane-bound APX activity was assessed using the method described by Kameoka *et al*. (2021), and DHAR and MDAR activities were measured as outlined by Tanaka *et al*. (2021).

GGP activity was measured according to Hamada *et al*. (2023). Arabidopsis leaves (0.2 g) frozen in liquid nitrogen were ground and homogenized in 1 ml of extraction buffer containing 50 mM Tris–HCl (pH 7.5), 10% glycerol, 2 mM DTT, 10 mM MgCl_2_, 1 mM aminocaproic acid, 1 mM benzamidine, and 1 mM phenylmethanesulfonylfluoride. After centrifugation (15 300*×g*) for 20 min at 4 °C, the supernatant was concentrated by ultrafiltration (15 300*×g*, 30 min, 4 °C) using an Amicon Ultra centrifugal filter (10 kDa, NMWL) and used for the following assay. The extract (20 µl) was added to 80 µl of a reaction mixture containing 50 mM Tris–HCl (pH 7.5), 2 mM MgCl_2_, 10 mM KCl, 1 mM dithiothreitol, and 100 µM GDP-D-glucose with or without 5 mM sodium phosphate, and incubated for 1 h at 26 °C. The reaction was stopped by boiling for 5 min, and the mixture was passed through 0.22 µm filters (Merck Millipore). The samples were injected into a UFLC system (Prominence UFLC, Shimadzu) equipped with a C-18 column (LUNA C18(2), Column 150×4.6 nm, Shimadzu). The mobile phase consisted of 50 mM phosphate buffer (pH 6.7), 5 mM tetrabutylammonium dihydrogen phosphate, 5 mg l^−1^ EDTA, and 3.5% methanol, and the flow rate was 1 ml min^−1^. GDP-D-glucose was detected at 254 nm using the SPD-20A UV-Vis detector (Shimadzu). Because GGP activity requires inorganic phosphate (Pi), Pi-independent degradation was subtracted from the Pi-dependent decrease in the substrate to calculate GDP-D-glucose degradation activity.

### RNA-seq analysis

Two-week-old wild-type, *vtc2-4*, and *vtc3-3* seedlings, grown on half-strength MS medium supplemented with 1% sucrose under the conditions described above, were incubated in darkness for 2 d, followed by a 6 h exposure to growth light (100 µmol photons m^−2^ s^−1^). Total RNA was extracted from shoot tissues using RNAiso Plus (Takara, Japan) and purified with NucleoSpin RNA Plus (Takara). RNA was fragmented and converted to double-stranded DNA using the TruSeq RNA Sample Preparation Kit v2 (Illumina). Library quality and quantity were assessed using a Bioanalyzer (Agilent) and a KAPA Library Quantification Kit (KAPA Biosystems), respectively. Sequencing was performed using an Illumina MiSeq with the MiSeq Reagent Kit v3 (300 cycles; Illumina). RNA-seq data were obtained from triplicate biological samples (*n*=3 per group) and statistically analyzed. Read mapping, data processing, and statistical analyses were conducted using the CLC Genomics Workbench (version 9.5.2). The gene expression dataset has been deposited in the Gene Expression Omnibus under accession number GSE293768. Gene Ontology (GO) enrichment analysis was performed using agriGO (version 2; Tian *et al*., 2017).

### Chlorophyll fluorescence measurements

Chlorophyll fluorescence measurements were performed using a Closed FluorCam 800MF (Photon Systems Instruments). Plants were dark-adapted for 30 minutes before measurements. Chlorophyll fluorescence parameters, *F*_v_/*F*_m_ and non-photochemical quenching (NPQ), were calculated according to Baker (2008) using the following equations:


$$\begin{array}{r} \;F_{v}/F_{m}=(F_{m}-F_{o})/F_{m},\;\mathrm{and}\\ \;\mathrm{NPQ}=(F_{m}\text{–}{F^{\prime }}_{m})/{F^{\prime }}_{m} \end{array}.$$

NPQ was measured under actinic light at an intensity of 90 µmol photons m^−2^ s^−1^.

### Accession numbers

VTC2, At4g26850; VTC3, At2g40860.

### Data analysis

The statistical methods used are described in the respective figure legends (Tukey–Kramer, Dunnett’s test, or Student’s *t*-test). Statistical calculations were performed using at least three independent biological replicates (see figure legends for details).

## Results

### VTC3 is required for ascorbate accumulation under high-light stress

**Fig. 1.**
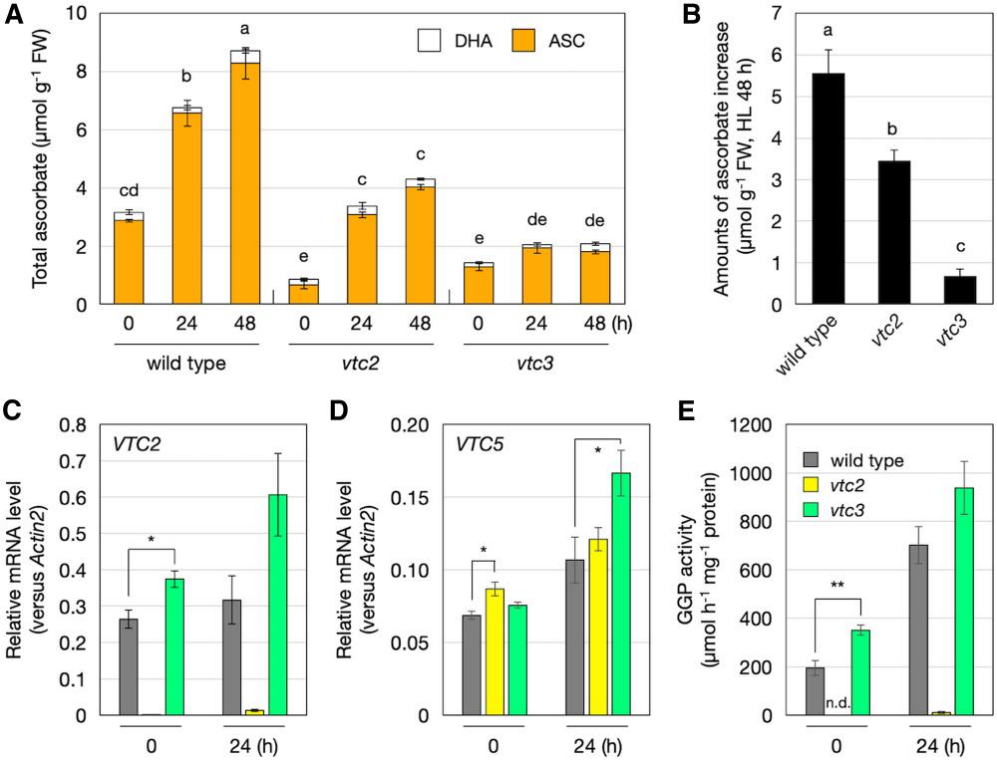
Ascorbate accumulation and GGP expression/activity in *vtc3-3* under high light. Wild-type, *vtc2-4*, and *vtc3-3* plants were grown in soil under low-light (40–60 μmol photons m^−2^ s^−1^) conditions for 3 weeks and then exposed to high-light stress (1500 μmol photons m^−2^ s^−1^) for 48 h. Fully expanded leaves were used in subsequent experiments. (A) Total ascorbate concentration, comprising the sum of reduced ascorbate (ASC) and dehydroascorbate (DHA) concentrations. (B) Increase in ascorbate concentration from before to after high-light exposure (48 h). In (A, B), data are presented as the mean ±SE of four biological replicates. Different letters indicate significant differences (*P*<0.05; one- or two-way ANOVA followed by Tukey–Kramer test). (C, D) Transcript levels of (C) *VTC2* and (D) *VTC5* determined using qPCR analysis. (E) GGP activity determined as GDP-D-glucose degrading activity. Data are presented as the mean ±SE of three biological replicates. Asterisks indicate significant differences between the wild type and mutant lines (*P<0.05, **P<0.01; Student’s *t*-test). In (C, E), because *VTC2* transcript levels and GGP activity were negligible in *vtc2-4*, statistical analysis was performed only between the wild type and *vtc3-3*. FW, fresh weight.

To evaluate the role of VTC3 in ascorbate accumulation under high-light conditions, we used a T-DNA insertion mutant for the *VTC3* gene, referred to hereafter as *vtc3-3* (Conklin *et al*., 2013), as well as the *vtc2-4* mutant (Lim *et al*., 2016). In *vtc3-3*, the T-DNA is inserted in the seventh exon of *VTC3*, whereas in *vtc2-4*, the insertion occurs in the third exon of *VTC2*. Both mutants have been previously characterized as null alleles lacking full-length transcripts of the respective genes (Conklin *et al*., 2013; Lim *et al*., 2016). This was further confirmed by our RT–PCR analysis (Supplementary Fig. S2). Under low-light conditions, *vtc2-4* and *vtc3-3* mutants exhibited approximately 26% and 45% of wild-type ascorbate concentrations, respectively (Fig. 1A), consistent with previous reports (Conklin *et al*., 2013; Lim *et al*., 2016). Exposure to high-light stress induced a time-dependent increase in ascorbate content in wild-type plants. In contrast, the *vtc3-3* mutant displayed only a minimal and statistically insignificant increase in ascorbate concentration. The ascorbate pool size in *vtc2-4* remained significantly lower than in wild-type plants under both low-light and high-light conditions. However, high-light stress markedly increased the ascorbate concentration in *vtc2-4* (Fig. 1A), corroborating previous findings (Terai *et al*., 2020; Hamada *et al*., 2023). The elevated concentrations of ascorbate after 48 h of high-light exposure were approximately 5.5 µmol g^−1^ fresh weight (FW) in the wild type, 3.4 µmol g^−1^ FW in *vtc2-4*, and 0.7 µmol g^−1^ FW in *vtc3-3* (Fig. 1B). Neither the *vtc2-4* nor the *vtc3-3* mutation affected the ascorbate redox state under either low- or high-light conditions. These results demonstrate that VTC3 is essential for the accumulation of ascorbate in response to high-light exposure.

### VTC3 is not required for VTC2 expression or GGP enzyme activity

To test our first hypothesis—that VTC3 regulates the light-dependent activation of VTC2/GGP—we analyzed the transcript levels of *VTC2* and *VTC5* before and after 24 h of high-light exposure. In *vtc2-4* mutants, *VTC2* transcripts were nearly undetectable (Fig. 1C). In wild-type plants, the transcript levels of both *VTC2* and *VTC5* showed a modest increase in response to high-light stress. Intriguingly, these increases were more pronounced in *vtc3-3* mutants. This up-regulation of *VTC2* and *VTC5* in *vtc3-3* mutants may represent a compensatory response to ascorbate deficiency. However, no similar increase in *VTC5* expression was observed in *vtc2-4* mutants after high-light exposure (Fig. 1D), possibly because *vtc2-4* mutants retained higher ascorbate concentrations than *vtc3-3* mutants under high-light conditions (Fig. 1A).

To assess VTC2 protein levels, we obtained three anti-VTC2 antibodies targeting different VTC2 peptides. Unfortunately, none of these antibodies successfully detected VTC2 via western blotting, likely due to low antibody specificity or the very low abundance of VTC2 protein. As an alternative, we measured GGP activity using a Pi-dependent GDP-D-glucose degradation assay, a reliable indicator of endogenous GGP activity (Hamada *et al*., 2023). In wild-type plants, GGP activity increased in response to high-light exposure, whereas in *vtc2-4* mutants, activity was nearly undetectable (Fig. 1E). Notably, the absence of VTC3 did not reduce GGP activity; rather, it appeared to enhance it, consistent with the observed increase in *VTC2* and *VTC5* transcript levels in *vtc3-3* mutants. Contrary to our hypothesis, these results indicate that VTC3 is not essential for the expression and activity of GGP.

### VTC3 does not affect ascorbate redox enzyme activities or transcriptome profiles

**Fig. 2.**
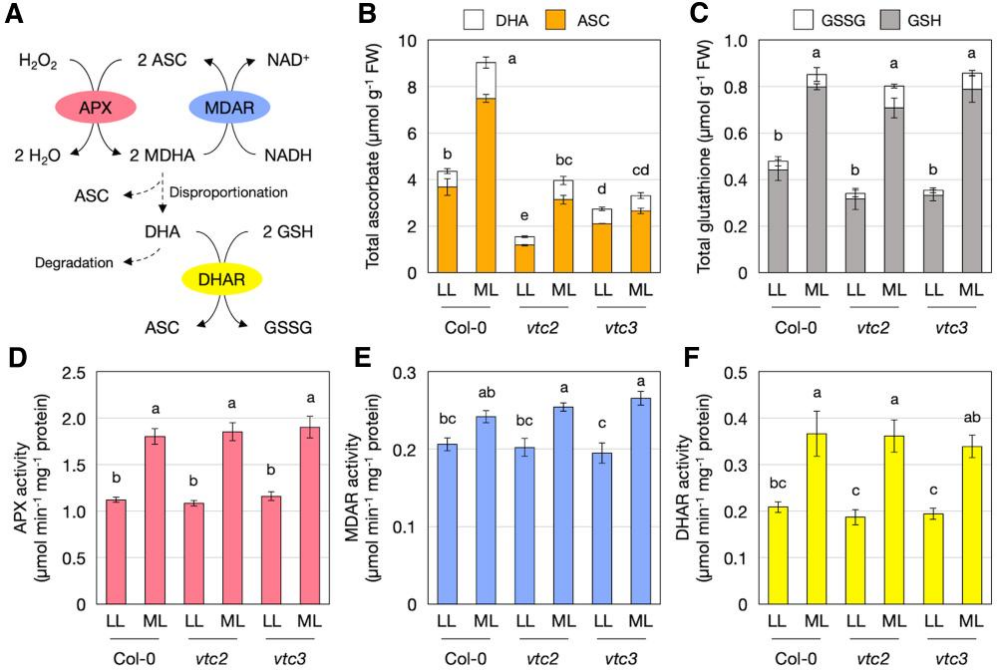
Antioxidant concentrations and enzyme activities related to ascorbate redox turnover in *vtc3-3* under high light. Wild-type, *vtc2-4*, and *vtc3-3* plants were grown on MS plates under low-light (40–60 μmol photons m^−2^ s^−1^) conditions for 2 weeks and then exposed to moderate-light stress (650 μmol photons m^−2^ s^−1^) for 24 h. (A) Ascorbate oxidation by ascorbate peroxidase (APX) and its recycling through monodehydroascorbate reductase (MDAR) and dehydroascorbate reductase (DHAR). ASC, reduced ascorbate; DHA, dehydroascorbate; GSH, reduced glutathione; GSSG, oxidized glutathione. (B) Total ascorbate content (sum of ASC and DHA contents). (C) Total glutathione content (sum of GSH and GSSG contents). (D) APX activity. (E) MDAR activity. (F) DHAR activity. Data are presented as the mean ±SE of three or four biological replicates. Different letters indicate significant differences (*P*<0.05; two-way ANOVA followed by Tukey–Kramer test). FW, fresh weight.

We next speculated that VTC3 may contribute to ascorbate redox cycling rather than biosynthesis. To test this possibility, we examined the impact of VTC3 on glutathione concentrations and the activities of APX, DHAR, and MDAR. Plants grown on agar plates were used for these experiments. Two-week-old wild-type, *vtc2-4*, and *vtc3-3* plants on agar plates were exposed to moderate-light stress (650 µmol photons m^−2^ s^−1^) for 24 h. This stress increased the ascorbate concentration in both wild-type and *vtc2-4* plants, but this response was nearly absent in *vtc3-3* mutants, consistent with the data obtained from *in vivo* soil experiments (Fig. 2).

The size of the glutathione pool increased under moderate-light stress (650 µmol photons m^−2^ s^−1^) in all genotypes, with no significant differences observed among the wild type, *vtc2-4*, and *vtc3-3*. Additionally, APX, DHAR, and MDAR activities were up-regulated under these conditions, but neither the *vtc2-4* nor the *vtc3-3* mutation influenced their activities under either low-light or moderate-light stress conditions (Fig. 2). These findings suggest that VTC3 does not significantly affect ascorbate redox cycling.

**Fig. 3.**
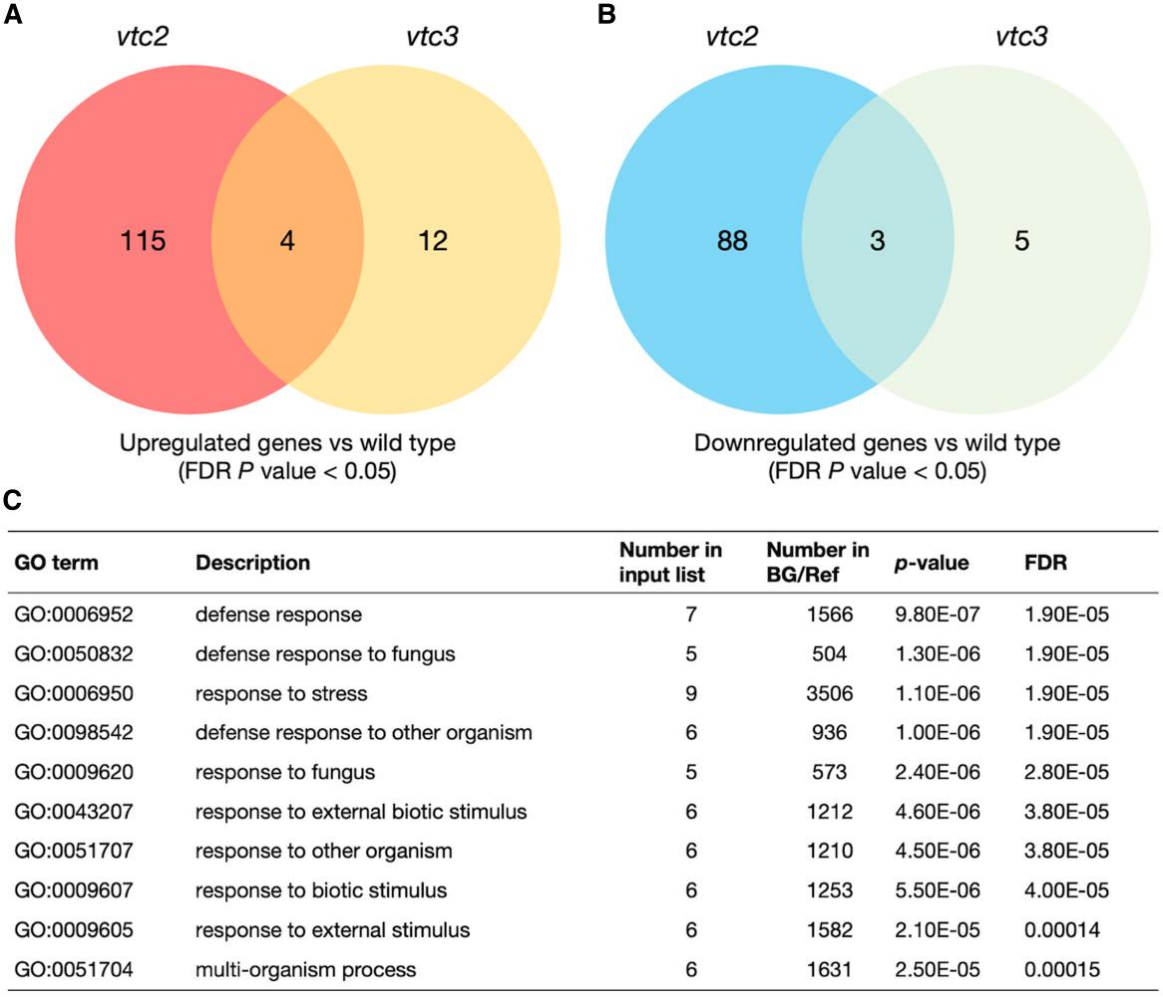
Transcriptomic changes in *vtc2-4* and *vtc3-3*. Wild-type, *vtc2-4*, and *vtc3-3* plants were grown on MS plates under low-light (40–60 μmol photons m^−2^ s^−1^) conditions for 2 weeks and then incubated in darkness for 2 d (to reduce the ascorbate pool size), followed by a 6 h exposure to light (100 µmol photons m^−2^ s^−1^). From the triplicate data, we selected the genes whose expression was significantly up-regulated (false discovery rate <0.05) in *vtc2-4* or *vtc3-3* compared with the wild-type plants. (A, B) Venn diagrams showing the overlap between sets of genes up-regulated (A) or down-regulated (B) in *vtc2-4* and *vtc3-3*. (C) Gene Ontology (GO) enrichment analysis (biological process) of 12 genes whose expression was up-regulated in *vtc3-3* compared with the wild-type plants. (C) GO terms enriched in genes whose expression was specifically promoted in *vtc3-3*. BG, background; Ref, reference.

To further investigate the function of VTC3, we performed transcriptome analysis. For this experiment, a drastic light/dark shift was used instead of light stress to induce a stronger response. Two-week-old wild-type, *vtc2-4*, and *vtc3-3* plants grown on agar plates under low-light conditions were incubated in darkness for 2 d, followed by a 6 h exposure to growth light (100 µmol photons m^−2^ s^−1^). Differentially expressed genes (DEGs) (false discovery rate <0.05) were identified from triplicate datasets by comparing the mutants with wild-type plants (Fig. 3; Supplementary Table S2). In total, over 200 genes were differentially expressed in *vtc2-4* mutants, whereas only 24 genes, including *VTC3* itself, were affected in *vtc3-3* mutants. Among these, seven genes were commonly affected in both mutants. The relatively minor transcriptomic impact of VTC3 compared with VTC2 likely reflects the weaker ascorbate-deficient phenotype of *vtc3-3*. GO enrichment analysis showed that ‘defense response to pathogens’ was the most enriched GO term among the 12 genes up-regulated in *vtc3* mutants (Fig. 3). Furthermore, down-regulated genes specific to *vtc3-3* included cytochrome P450 superfamily protein (AT3G44970), transmembrane protein (AT5G01881), mto1 responding down 1 (AT1G53480), and phosphoinositide 4-kinase gamma 1 (AT2G40850), none of which are known to be directly associated with ascorbate metabolism (Supplementary Table S2). These results suggest that VTC3 plays a more limited role in transcriptomic regulation than VTC2.

### Simultaneous loss of VTC2 and VTC3 exacerbates ascorbate deficiency and increases light-stress sensitivity

**Fig. 4.**
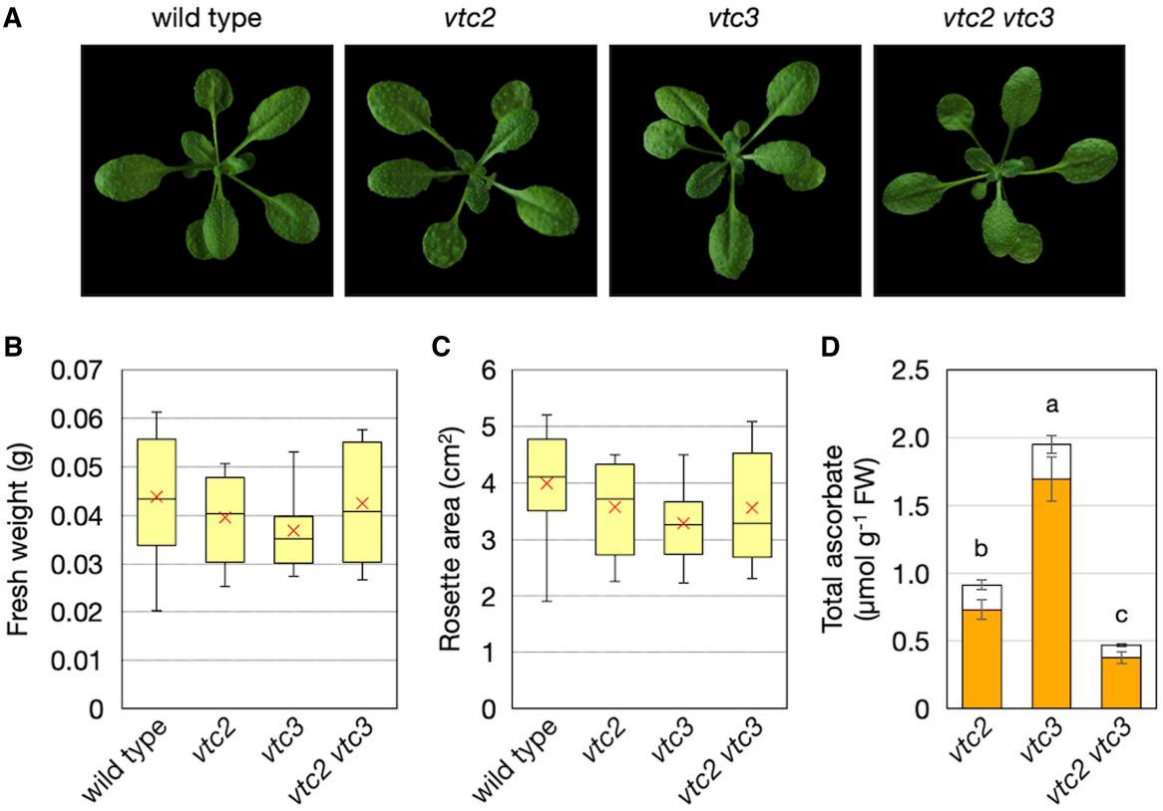
Phenotypes and ascorbate profiles of *vtc2-4 vtc3-3* double mutants. Wild-type, *vtc2-4*, *vtc3-3*, and *vtc2-4 vtc3-3* plants were grown in soil under low-light (40–60 μmol photons m^−2^ s^−1^) conditions for 3 weeks. Fully expanded leaves were used for ascorbate measurements. (A) Representative phenotypes of plants. (B, C) Box plots illustrating fresh weights (B) and rosette area (C). Horizontal bars and crosses within the boxes represent the median and mean values from 12 biological replicates, respectively, and boxes represent the 25th and 75th percentiles. The outer error bars (whiskers) represent maximum and minimum values. There was no significant difference between genotypes (one-way ANOVA). (D) Total ascorbate concentration, comprising the sum of reduced ascorbate (ASC) and dehydroascorbate (DHA) concentrations. Data are presented as the mean ±SE of five biological replicates. Different letters indicate significant differences (*P*<0.05; one-way ANOVA followed by Tukey–Kramer test). FW, fresh weight.

To test our second hypothesis that the simultaneous loss of VTC2 and VTC3 would result in additive or synergistic effects on ascorbate pool size, we generated *vtc2-4 vtc3-3* double mutants and analyzed them under both low- and high-light conditions. Homozygous double mutants were identified by genotyping the F_2_ population following crossing (Supplementary Fig. S2). Compared with single mutants, the double mutants exhibited the lowest ascorbate concentrations, retaining only approximately 51% of the ascorbate content observed in *vtc2-4* single mutants, equivalent to ∼14% of wild-type concentrations (Fig. 4). Notably, except for mutants entirely lacking detectable ascorbate (e.g. *vtc2 vtc5* double mutants), *vtc2-4 vtc3-3* double mutants displayed the lowest ascorbate concentrations reported among ascorbate-deficient mutants. Despite these extremely low concentrations, their growth under low-light conditions for 3 weeks was comparable to that of wild-type plants and single mutants (Fig. 4), suggesting that even 14% of wild-type ascorbate concentrations is sufficient to support Arabidopsis growth under controlled laboratory conditions.

**Fig. 5.**
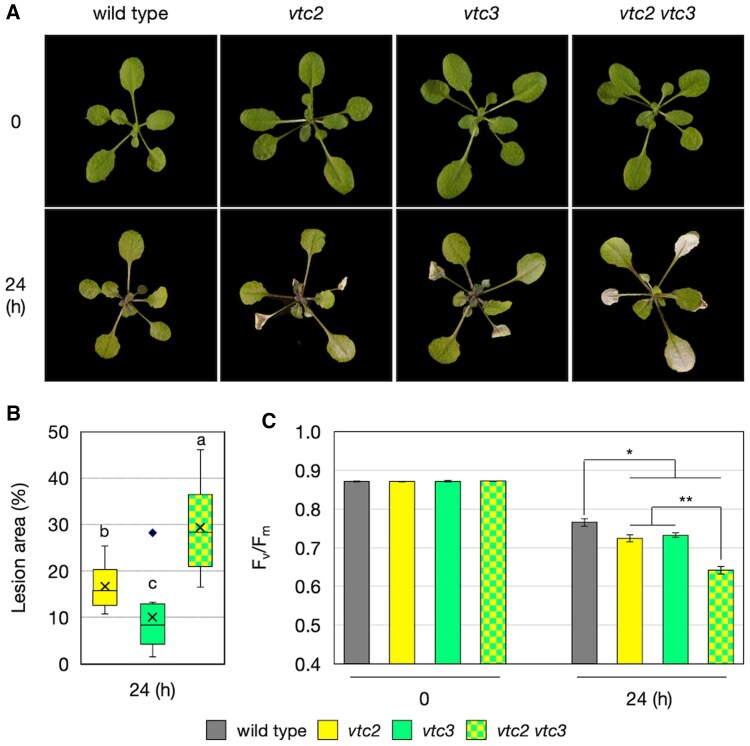
Sensitivity of *vtc2-4 vtc3-3* double mutants to high-light stress. Wild-type, *vtc2-4*, *vtc3-3*, and *vtc2-4 vtc3-3* plants were grown in soil under low-light (40–60 μmol photons m^−2^ s^−1^) conditions for 3 weeks and then exposed to high-light stress (1500 μmol photons m^−2^ s^−1^) for 24 h. (A) Representative phenotypes of plants before and after high-light stress. (B) Box plots illustrating the lesion area (% of whole rosette area). Data were obtained from nine plants per genotype in three independent experiments. As wild-type plants showed no lesions, lesion area analysis was not performed. Horizontal bars and crosses within the boxes represent the median and mean values from five biological replicates, respectively, and boxes represent the 25th and 75th percentiles. The outer error bars (whiskers) represent the maximum and minimum values, and the diamond represents an outlier. Different letters indicate significant differences (*P*<0.05; one-way ANOVA followed by Tukey–Kramer test). (C) The maximum quantum yield of photosystem II (F_v_/F_m_) before and after high-light stress. Asterisks indicate significant differences between genotypes (*P<0.05, **P<0.01; one-way ANOVA followed by Tukey–Kramer test).

When exposed to high-light stress for 24 h, *vtc2-4* and *vtc3-3* single mutants exhibited photobleaching, primarily in older leaves (Fig. 5). In contrast, the *vtc2-4 vtc3-3* double mutants displayed a more severe photobleaching phenotype, extending to mature leaves, and exhibited the most pronounced cell death, consistent with their extremely low ascorbate content. Reflecting these phenotypes, the reduction in the maximum quantum yield of photosystem II (F_v_/F_m_) under high-light stress was most pronounced in the double mutants (Fig. 5), indicating severe damage to photosynthesis. To test whether the heightened light sensitivity of the double mutants is attributable to ascorbate deficiency, we conducted a chemical complementation experiment using sodium ascorbate. Detached leaves from 3-week-old *vtc2-4 vtc3-3* plants were floated on buffer with or without 10 mM sodium ascorbate under continuous light for 24 h, followed by 24 h of high-light exposure. Under these conditions, clear leaf bleaching and intense trypan blue staining were observed only in *vtc2-4 vtc3-3* leaves without sodium ascorbate treatment. Notably, these symptoms were completely abolished by sodium ascorbate treatment (Supplementary Fig. S3), indicating that the severe photooxidative damage in the double mutants is primarily due to their profound ascorbate deficiency.

**Fig. 6.**
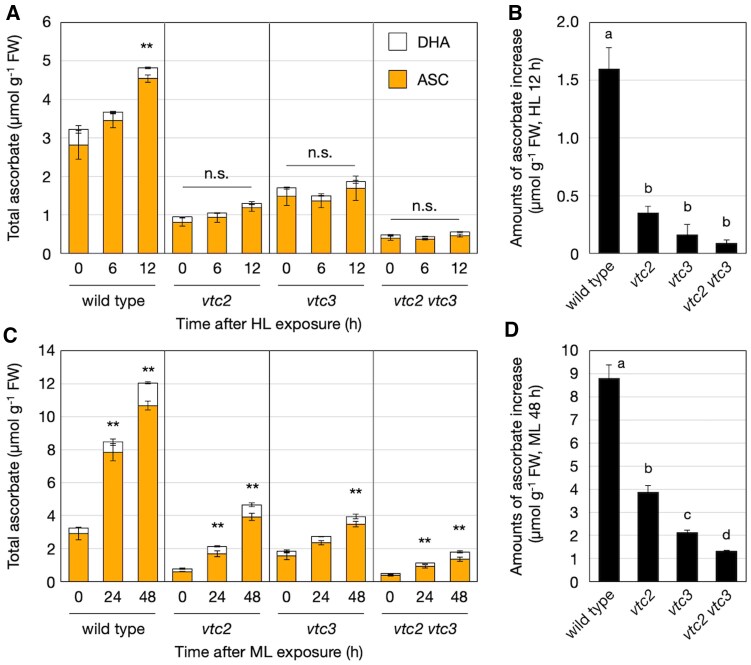
Ascorbate profiles in *vtc2-4 vtc3-3* under high-light (HL) or moderate-light (ML) stress. Wild-type, *vtc2-4*, *vtc3-3*, and *vtc2-4 vtc3-3* plants were grown in soil under low-light (40–60 μmol photons m^−2^ s^−1^) conditions for 3 weeks and then exposed to high-light stress (1500 μmol photons m^−2^ s^−1^) for 24 h (A, B), or to moderate-light stress (750 μmol photons m^−2^ s^−1^) for 48 h (C, D). Fully expanded leaves were used for ascorbate measurements. (A, C) Total ascorbate concentration, comprising the sum of reduced ascorbate (ASC) and dehydroascorbate (DHA) concentrations. Data are presented as the mean ±SE of three or four biological replicates. Significant differences between data before (0 h) and after light stress for each genotype are indicated with asterisks ( **P<0.01; one-way ANOVA with Dunnett’s *post hoc* test for total content). (B, D) Increases in the concentrations of ascorbate from before to after HL or ML exposure (24 h or 48 h, respectively). Data are presented as the mean ±SE of three or four biological replicates. Different letters indicate significant differences (*P*<0.05; one-way ANOVA followed by Tukey–Kramer test). FW, fresh weight; n.s., not significant.

Considering the heightened sensitivity of the *vtc2-4 vtc3-3* mutants to high-light stress, we measured the ascorbate concentrations in wild-type plants and mutants during the early stages of high-light exposure (6 h and 12 h). The ascorbate concentrations in wild-type plants and *vtc2-4* mutants increased approximately 1.5-fold by 12 h after high-light exposure (Fig. 6), but there was no statistically significant difference. Furthermore, no significant increase in ascorbate concentrations was observed in *vtc3-3* or *vtc2-4 vtc3-3* mutants. However, the early-stage increase in ascorbate in wild-type plants and *vtc2-4* mutants was relatively modest (Fig. 6), making detailed comparisons between mutants challenging.

To further investigate the ascorbate accumulation response under different light intensities, we conducted similar experiments under moderate-light conditions (750 µmol photons m^−2^ s^−1^). Under this moderate-light stress, ascorbate accumulation was observed even in *vtc3-3* and *vtc2-4 vtc3-3* mutants; however, the increase was significantly smaller compared with *vtc2-4*, with the double mutants exhibiting the lowest stress-induced ascorbate accumulation (Fig. 6). These results demonstrate that the simultaneous knockout of VTC2 and VTC3 severely limits both steady-state ascorbate concentrations and stress-induced ascorbate accumulation, thereby dramatically increasing sensitivity to photooxidative stress.

### Impaired induction of non-photochemical quenching in vtc2-4 vtc3-3 double mutants

**Fig. 7.**
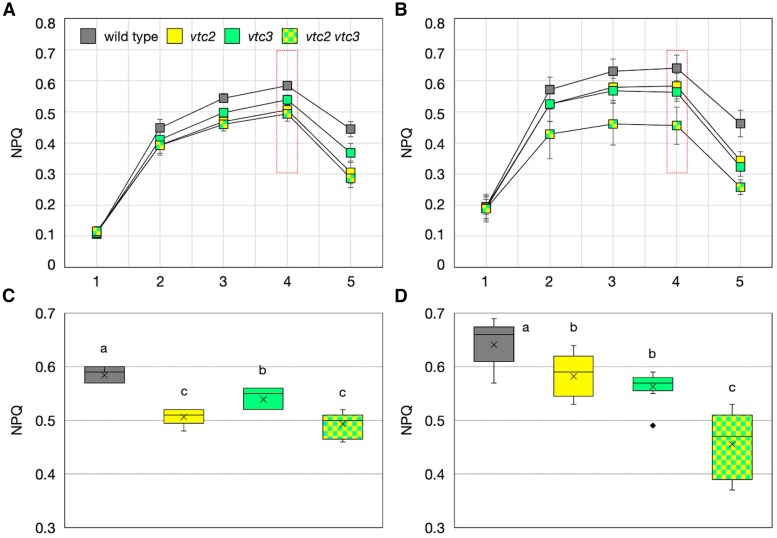
Non-photochemical quenching (NPQ) in *vtc2-4 vtc3-3* before and after high-light stress. Wild-type, *vtc2-4*, *vtc3-3*, and *vtc2-4 vtc3-3* plants were grown in soil under low-light (40–60 μmol photons m^−2^ s^−1^) conditions for 3 weeks and then exposed to high-light stress (1500 μmol photons m^−2^ s^−1^) for 24 h. (A, B) NPQ induction upon the onset of actinic light (90 µmol photons m^−2^ s^−1^) before (A) and after (B) stress. Data are presented as the mean ±SE of three or four biological replicates. The red dashed boxes indicate the data points used for statistical analysis. (C, D) Box plots illustrating NPQ data before (C) and after (D) stress, corresponding to the areas outlined with red dashed boxes in (A) and (B), respectively. Horizontal bars and crosses within the boxes represent the median and mean values from 12 biological replicates, respectively, and boxes represent the 25th and 75th percentiles. The outer error bars (whiskers) represent maximum and minimum values, and the diamond represents an outlier. Different letters indicate significant differences (*P*<0.05; one-way ANOVA followed by Tukey–Kramer test).

As ascorbate is implicated in the induction of NPQ (Müller-Moulé *et al*., 2004), a photoprotective mechanism that dissipates excess absorbed light energy as heat, we investigated how the severe ascorbate deficiency in *vtc2-4 vtc3-3* double mutants affects NPQ. Plants were grown under low-light conditions for 3 weeks before being subjected to high-light stress for 24 h. Under low-light conditions, NPQ induction was partially suppressed in all mutants compared with wild-type plants, with statistically significant but relatively small differences (Fig. 7A, C). Among the single mutants, *vtc2-4* exhibited a greater reduction in NPQ induction compared with *vtc3-3*, consistent with its lower ascorbate pool size. Although the *vtc2-4 vtc3-3* double mutants showed a trend toward further reduced NPQ values, the difference was not statistically significant compared with *vtc2-4* single mutants. Following high-light exposure, NPQ suppression was most pronounced in *vtc2-4 vtc3-3* double mutants, with NPQ being significantly lower than in either *vtc2-4* or *vtc3-3* single mutants (Fig. 7B, D). Interestingly, although *vtc2-4* exhibited lower NPQ values than *vtc3-3* under low-light conditions (Fig. 7C), this difference disappeared under high-light stress (Fig. 7D). These variations in NPQ induction closely paralleled the differences in ascorbate pool sizes among the mutants. These findings provide new insights into the relationship between NPQ induction and ascorbate concentrations, highlighting the additive effects of *vtc2-4* and *vtc3-3* mutations on NPQ suppression under high-light stress. The results suggest that ascorbate plays a critical role in NPQ induction under both low- and high-light conditions and that VTC2 and VTC3 contribute to this regulation through their roles in ascorbate accumulation.

## Discussion

Among the *vtc* mutant series, *vtc3* is the only mutant with a mutation in a gene that does not encode a biosynthetic enzyme (Conklin *et al*., 2013). VTC3 is a unique protein containing both protein kinase and phosphatase domains and is essential for ascorbate accumulation in response to continuous exposure to growth light. Given that VTC2/GGP is the rate-limiting enzyme in ascorbate biosynthesis and that its expression and activity are enhanced by light, we initially hypothesized that VTC3 regulates ascorbate biosynthesis at the VTC2/GGP step. However, this hypothesis was strongly refuted, as the loss of VTC3 did not reduce *VTC2/VTC5* transcription or GGP activity (Fig. 1). As previously reported, VTC2 translation is controlled by ascorbate via an open reading frame in the 5′-untranslated region of the *VTC2* gene (Laing *et al*., 2015). Because we were unable to examine VTC2/GGP expression at the protein level, we cannot entirely rule out the possibility that VTC3 influences VTC2 translation. However, if this were the case, the absence of VTC3 would likely lead to reduced GGP activity, which was not observed (Fig. 1). Thus, we conclude that VTC3 is not required for the expression and activity of VTC2/GGP.

We also found that the loss of VTC3 did not affect glutathione profiles or the activities of APX, DHAR, and MDAR (Fig. 2). Since we did not examine the expression or activity of individual isoforms of these enzymes, we cannot rule out the possibility that VTC3 affects ascorbate redox cycling in specific compartments. However, recent studies have demonstrated that the complementary actions of MDARs, DHARs, and glutathione maintain a robust ascorbate recycling system in Arabidopsis (Maruta *et al*., 2024). For instance, the loss of all three *DHAR* genes has a negligible impact on ascorbate contents, even under stress conditions (Rahantaniaina *et al*., 2017; Terai *et al*., 2020). Considering the resilience of the ascorbate recycling system, it is unlikely that VTC3 plays a role in regulating ascorbate redox cycling. Another possible function of VTC3 could be the inhibition of ascorbate degradation. However, this is also unlikely, as ascorbate degradation begins with DHA, and ascorbate recycling capacity was unaffected in *vtc3* mutants.

Since VTC3 contains both protein kinase and phosphatase domains, it was also plausible that it mediates light-dependent signaling pathways to enhance ascorbate pool size. However, transcriptome analysis revealed that only a limited number of genes were specifically up- or down-regulated in *vtc3-3* mutants (Fig. 3), and none had known associations with ascorbate metabolism. However, one of the genes down-regulated in *vtc3-3* mutants encodes phosphoinositide 4-kinase gamma 1 (PI4Kγ1). Although this gene has not been directly linked to ascorbate metabolism, it may affect the pool size of *myo*-inositol, a potential precursor of ascorbate in the alternative biosynthetic pathway (Lorence *et al*., 2004). While the physiological relevance of this pathway in Arabidopsis remains under debate (Maruta, 2022), the potential role of PI4Kγ1 in regulating ascorbate concentrations through *myo*-inositol metabolism warrants further investigation. GO enrichment analysis identified ‘defense response to pathogens’ as the most enriched term among the 12 genes whose expression was specifically enhanced in *vtc3* mutants (Fig. 3; Supplementary Table S2). This finding aligns with previous observations in *vtc1* mutants, in which ascorbate deficiency was linked to enhanced pathogen responses (Pastori *et al*., 2003). Thus, the transcriptional activation of some defense-related genes in *vtc3-3* mutants is likely a secondary effect of ascorbate deficiency rather than associated with the direct involvement of VTC3 in defense signaling. In *vtc2-4* mutants, 115 genes were up-regulated (Fig. 3), with GO terms related to ‘response to salicylic acid’ and ‘response to chitin’ being enriched (Supplementary Table S2), further suggesting a link between ascorbate deficiency and pathogen responses.

In our RNA-seq analysis, we identified 210 DEGs in the *vtc2-4* mutant (Fig. 3). This number is substantially lower than the 963 DEGs previously reported in *vtc2* mutants by Kerchev *et al.* (2011), who used microarray analysis. This discrepancy is likely due to differences in experimental conditions. In our study, 2-week-old seedlings were grown aseptically on agar plates under long-day conditions, followed by 48 h of darkness and a 6 h light treatment before sampling. In contrast, Kerchev *et al*. (2011) used fully mature 6-week-old plants grown in soil under short-day conditions. These differences in plant age, growth medium, and light regime likely contributed to the differing transcriptome responses. Moreover, only 11 out of the 119 up-regulated genes in *vtc2-4* overlapped with the 378 up-regulated genes in the *vtc2* dataset from Kerchev *et al*. (2011) (Supplementary Table S2), suggesting the condition-specific nature of transcriptomic responses to ascorbate deficiency. However, both studies consistently observed an increase in the expression of pathogen-response-related genes, suggesting that this may be a core feature of ascorbate deficiency. Since the transcriptomic consequences of ascorbate deficiency remain incompletely understood, further comparative studies under standardized conditions will be important to clarify its context-dependent effects.

Taken together, these findings indicate that the loss of VTC3 has minimal impact on VTC2/GGP, ascorbate redox cycling, or the transcriptome. Nevertheless, the impaired light-induced ascorbate accumulation observed in *vtc3-3* mutants suggests that VTC3 may regulate ascorbate biosynthesis at steps other than VTC2/GGP. One plausible possibility is that VTC3 influences enzymes upstream of VTC2 in the D-Man/L-Gal pathway, such as PMI, PMM, GMP, and GME. These enzymes are responsible for synthesizing GDP-L-galactose, the direct substrate of VTC2/GGP, and reduced activity in any of these steps could limit VTC2 function despite unaltered transcript levels and enzyme activity. Alternatively, VTC3 may affect the supply of essential cofactors required for the optimal activity of VTC2/GGP or its upstream enzymes. For example, VTC2/GGP activity is dependent on the availability of Pi. Further studies will be required to identify the precise molecular targets of VTC3 and to clarify how it contributes to the light-responsive regulation of ascorbate biosynthesis. Given that *VTC3* encodes a putative kinase/phosphatase, it may also play broader regulatory roles beyond ascorbate biosynthesis.

Consistent with previous reports (Terai *et al*., 2020 ; Hamada *et al*., 2023), *vtc2-4* mutants retained the ability to increase ascorbate accumulation in response to light stress (Fig. 1). We initially hypothesized that this ability might be due to the activation of residual VTC5. However, *VTC5* transcription showed little to no increase in *vtc2-4* mutants compared with the wild type, and GGP activity was nearly undetectable in *vtc2-4* mutants even under high-light stress (Fig. 1). This could be due to GGP activity in *vtc2-4* being below the detection limit. Indeed, the simultaneous loss of VTC2 and VTC5 results in ascorbate concentrations below the detection limit, indicating that VTC5 functions as an active enzyme in ascorbate biosynthesis (Dowdle *et al*., 2007 ; Lim *et al*., 2016). Alternatively, because we used GDP-D-glucose instead of GDP-L-galactose (the physiological substrate, which is commercially unavailable) to measure GGP activity, our assay may not fully reflect endogenous GGP activity. Nevertheless, it is intriguing that ascorbate concentrations in *vtc2* mutants can increase significantly without a measurable increase in GGP activity. If alternative biosynthetic pathways are not considered, this suggests the existence of a mechanism that enhances the GGP response independent of measurable GGP activity. The recently reported metabolon formation of ascorbate biosynthetic enzymes may be involved (Fenech *et al*., 2021), but further experimental validation is required.

Another key objective of this study was to examine the impact of the simultaneous loss of VTC2 and VTC3 on ascorbate pool size. As expected, *vtc2-4 vtc3-3* double mutants exhibited the lowest ascorbate concentrations reported among viable ascorbate-deficient mutants, retaining only ∼14% of wild-type concentrations (Fig. 4). Nevertheless, under low-light conditions, *vtc2-4 vtc3-3* double mutants displayed a healthy phenotype similar to wild-type plants. The original *vtc2-1* mutant, isolated via an ethyl methanesulfonate mutagenesis screen, has been reported to exhibit dwarfism under standard growth conditions, whereas the T-DNA insertion mutant *vtc2-4*, despite having comparable ascorbate concentrations, does not exhibit dwarfism (Lim *et al*., 2016). It has been suggested that the dwarfism observed in *vtc2-1* may be due to a mutation in another gene. The relationship between ascorbate concentrations and growth must be carefully evaluated, as it is highly influenced by growth light intensity. For instance, when grown under relatively higher light conditions (200 µmol photons m^−2^ s^−1^), both *vtc2-1* and *vtc2-4* exhibit similar degrees of dwarfism (Plumb *et al*., 2018). Considering this complexity, our data suggest that under artificial low-light conditions, concentrations of ascorbate as little as 14% of wild-type concentrations are sufficient for normal growth in Arabidopsis. This finding provides the idea that the primary reason plants accumulate high concentrations of ascorbate is to protect against oxidative stress rather than to fulfill its coenzyme functions.

The loss of VTC3 significantly impaired high-light-induced ascorbate accumulation in both wild-type and *vtc2-4* backgrounds (Fig. 6). *vtc2-4 vtc3-3* double mutants maintained extremely low ascorbate concentrations even under high-light conditions, leading to more severe photooxidative damage than in either of the single mutants (Fig. 5). In terms of photobleaching severity, *vtc2-4* was more sensitive to high light than *vtc3-3*, and the sensitivity of the double mutant was entirely consistent with its steady-state ascorbate pool size (Fig. 4). NPQ values were statistically significantly lower in all *vtc* mutants compared with wild-type plants under low-light conditions, with *vtc2-4* and the double mutant exhibiting greater reductions than *vtc3-3* (Fig. 7). However, no significant difference was observed between *vtc2-4* and the double mutant under low-light conditions, suggesting that NPQ suppression due to ascorbate deficiency may reach a threshold at ∼30% of wild-type ascorbate concentrations. Interestingly, this difference became more pronounced after high-light exposure, paralleling the fact that *vtc2-4* mutants still exhibited light-induced ascorbate accumulation, whereas this response was severely impaired in the double mutants (Fig. 6). So far, *vtc1* and *vtc4* mutants have also been used to study ascorbate deficiency, but these mutants have pleiotropic effects unrelated to ascorbate metabolism (Torabinejad *et al*., 2009; Barth *et al*., 2010). In contrast, *vtc2-4* and *vtc3-3* mutants provide a more targeted approach. Since the *vtc2-4 vtc3-3* double mutant retains the characteristics of both single mutants, comparative analysis of these mutants represents a powerful tool for elucidating the physiological roles of ascorbate.

In conclusion, our findings indicate that VTC3 is not required for the expression or activity of VTC2/GGP, ascorbate redox cycling (including ascorbate recycling), or the regulation of the transcriptome. These negative results provide important insight by excluding several plausible roles of VTC3 and thereby advancing our understanding of its physiological function. Although the fundamental function of VTC3 remains unresolved, our findings suggest that VTC3 regulates ascorbate biosynthesis at a step other than VTC2/GGP, and future research will focus on validating this notion. Furthermore, this study revealed that the simultaneous knockout of VTC2 and VTC3 exacerbates ascorbate deficiency and increases sensitivity to light stress, likely due to the combined loss of ascorbate biosynthesis capacity (via VTC2) and regulatory modulation (via VTC3), both of which are essential for maintaining sufficient ascorbate concentrations under photooxidative stress. The newly generated *vtc2-4 vtc3-3* double mutant retains only ∼14% of wild-type ascorbate concentrations. Given its distinct physiological characteristics, such as impaired NPQ and enhanced photooxidative stress sensitivity, this double mutant, in combination with the single mutants, provides a valuable genetic tool to further dissect the relationship between ascorbate concentrations and physiological responses. Notably, it represents the most severely ascorbate-deficient viable Arabidopsis mutant characterized to date, potentially enabling the discovery of previously unrecognized roles of ascorbate in plant physiology.

## Supplementary Material

eraf352_Supplementary_Data

## Data Availability

The data underlying this article are available in the article and its supplementary material. The raw and processed data of RNA-seq have been deposited in the Gene Expression Omnibus (https://www.ncbi.nlm.nih.gov/geo/) under accession number GSE293768.

## Supplementary data

The following supplementary data are available at *JXB* online.

Fig. S1. Expression stability of the reference gene *Actin2* across different genotypes and treatment conditions.

Fig. S2. Expression of *VTC2* and *VTC3* in *vtc* mutant lines.

Fig. S3. Ascorbate treatment alleviates light stress-induced cell death in the *vtc2-4 vtc3-3* double mutant

Table S1. List of primers used.

Table S2. List of genes whose expression is altered in *vtc* mutants and Gene Ontology enrichment analysis.

## Abbreviations

APX: ascorbate peroxidase
ASC: ascorbate
DHA: dehydroascorbate
DHAR: dehydroascorbate reductase
GGP: GDP-L-galactose phosphorylase
GME: GDP-D-mannose-3′,5′-epimerase
GMP: GDP-D-mannose pyrophosphorylase
GPP: L-galactose-1-phosphate phosphatase
GSH: reduced glutathione
L-GalDH: L-galactose dehydrogenase
L-GalLDH: L-galactono-1,4-lactone dehydrogenase
MDHA: monodehydroascorbate
MDAR: monodehydroascorbate reductase
NPQ: non-photochemical quenching
PMI: phosphomannose isomerase
PMM: phosphomannomutase
PP2C: protein phosphatase 2C
ROS: reactive oxygen species
VTC: vitamin C defective
